# Genetic Manipulation of Sirtuin 3 Causes Alterations of Key Metabolic Regulators in Melanoma

**DOI:** 10.3389/fonc.2021.676077

**Published:** 2021-04-16

**Authors:** Chandra K. Singh, Jasmine George, Gagan Chhabra, Minakshi Nihal, Hao Chang, Nihal Ahmad

**Affiliations:** ^1^ Department of Dermatology, University of Wisconsin, Madison, WI, United States; ^2^ William S. Middleton VA Medical Center, Madison, WI, United States

**Keywords:** melanoma, sirtuins, SIRT3, shRNA, cellular metabolism

## Abstract

The mitochondrial sirtuin SIRT3 plays key roles in cellular metabolism and energy production, which makes it an obvious target for the management of cancer, including melanoma. Previously, we have demonstrated that SIRT3 was constitutively upregulated in human melanoma and its inhibition resulted in anti-proliferative effects *in vitro* in human melanoma cells and *in vivo* in human melanoma xenografts. In this study, we expanded our data employing knockdown and overexpression strategies in cell culture and mouse xenografts to further validate and establish the pro-proliferative function of SIRT3 in melanocytic cells, and its associated potential mechanisms, especially focusing on the metabolic regulation. We found that short-hairpin RNA (shRNA) mediated SIRT3 knockdown in G361 melanoma cells showed diminished tumorigenesis in immunodeficient Nu/Nu mice. Conversely, SIRT3 overexpressing Hs294T melanoma cells showed increased tumor growth. These effects were consistent with changes in markers of proliferation (PCNA), survival (Survivin) and angiogenesis (VEGF) in xenografted tissues. Further, in *in vitro* culture system, we determined the effect of SIRT3 knockdown on glucose metabolism in SK-MEL-2 cells, using a PCR array. SIRT3 knockdown caused alterations in a total of 37 genes involved in the regulation and enzymatic pathways of glucose (32 genes) and glycogen (5 genes) metabolism. Functions annotation of these identified genes, using the ingenuity pathway analysis (IPA), predicted cumulative actions of decreased cell viability/proliferation, tumor growth and reactive oxygen species (ROS), and increased apoptosis in response to SIRT3 knockdown. Further, IPA gene network analysis of SIRT3 modulated genes revealed the interactions among these genes in addition to several melanoma-associated genes. Sirtuin pathway was identified as one of the top canonical pathways showing the interaction of SIRT3 with metabolic regulatory genes along with other sirtuins. IPA analysis also predicted the inhibition of HIF1α, PKM, KDM8, PPARGC1A, mTOR, and activation of P53 and CLPP; the genes involved in major cancer/melanoma-associated signaling events. Collectively, these results suggest that SIRT3 inhibition affects cellular metabolism, to impart an anti-proliferative response against melanoma.

## Introduction

Melanoma is the deadliest cancer of the skin, which arises from melanocytic cells that are derived from the neural crest and are primarily responsible for melanin production. Melanoma, if not treated early, possesses metastatic potential; and its incidence has been increasing over 30 years (1). Therefore, there is a critical need to develop novel mechanism-based strategies for an effective management of this neoplasm. Sirtuin-3 (SIRT3) is an important mitochondrial NAD^+^-dependent deacetylase that is known to target mitochondrial proteins for deacetylation and regulates a variety of cellular functions. Although SIRT3 is a mitochondrial protein, it is reported to move from mitochondria to nucleus under cellular stress (2). SIRT3 plays key roles in mitochondrial dynamics, metabolism and energy regulation, and therefore their possible roles in the progression of cancer are being intensively investigated (3). Studies have suggested that SIRT3 coordinates global shifts in mitochondrial activity by deacetylating proteins involved in diverse mitochondrial functions including energy metabolism and mitochondrial biogenesis (4, 5). It also plays important roles in the regulation of a variety of cellular processes, including transcription, insulin secretion, apoptosis and redox signaling (6). SIRT3 has also been implicated in multiple cutaneous functions including skin renewal, and response to environmental stressors (7, 8).

Based on a number of studies, SIRT3 has been shown to act either as a tumor suppressor or promoter depending on the cell milieus (9). For example, SIRT3 has been shown to be downregulated in gastric cancer (10), hepatocellular carcinoma (11), and pancreatic cancer (12). Conversely, higher expression of SIRT3 has been reported in cancers of esophagus (13), breast (14) and colon (15). The higher expression of SIRT3 has also been connected to therapy resistance (16) and poor prognosis (17), and its inhibition has been shown to enhance radiotherapeutic and chemotherapeutic drug cytotoxicity (18). We have recently demonstrated that SIRT3 was overexpressed in human melanoma, and its small hairpin RNA (shRNA) mediated knockdown provoked the senescence and growth inhibition in melanoma cells, *in vitro* and *in vivo* (19). In addition, forced overexpression of SIRT3 resulted in enhanced proliferation of melanocytic cells (19).

Cancer cells are known to be metabolically more active and consume high cellular fuel than normal cells (20). Thus, understanding the regulatory mechanisms associated with cellular energy metabolism at the gene level may be of fundamental importance that could be potentially explored for cancer treatment. Increasing evidence show that SIRT3 is required for the maintenance of cellular and mitochondria homeostasis through regulating mitochondrial metabolism and cellular redox balance system (6, 21, 22). In this study, we expanded our findings in additional experiments, employing knockdown and overexpression strategies in human melanoma cells and mouse xenografts to further validate the pro-proliferative function of SIRT3 in melanoma, and establish its associated potential mechanisms, especially focusing on metabolic regulation.

## Materials and Methods

### Cell Lines and Cell Culture

Human melanoma cell lines (SK-MEL-2, G361, and Hs294T), and human embryonic kidney cell line HEK293T were purchased from ATCC. SK-MEL-2 cells were maintained in Eagle’s minimal essential medium (supplemented with 1 mM sodium pyruvate and non-essential amino acids), G361 in McCoy’s 5a medium, and Hs294T and HEK293T cells in Dulbecco’s modified eagle’s medium with 10% fetal bovine serum at standard cell culture conditions (37°C, 5% CO_2_).

### Lentiviral SIRT3 Knockdown

SIRT3 knockdown was done in SK-MEL-2 and G361 cells and stable cell lines were established under puromycin selection, as described earlier (19). Briefly, for viral creation, HEK293T cells were transfected using calcium phosphate transfection method with empty vector pLKO.1 (SHC001V) and four different SIRT3 targeting short hairpin RNA (shRNA) procured from Sigma-Aldrich. Competent lentiviruses were harvested 48 h after transfection. The quality of lentivirus stock was assessed with Lenti-X GoStix (Clontech Laboratories). For transduction, viral media were added to SK-MEL-2 and G361 cells (at ~40% confluency) with 8 µg/ml of polybrene, 4-times over 2 days. On 3^rd^ day, cells were collected and SIRT3 shRNA constructs were checked for SIRT3 knockdown. The most efficient SIRT3 shRNA construct (TRCN0000038889) was used for generating SIRT3 knockdown stable cell lines by selection with puromycin (SK-MEL-2, 1.5 μg/ml for 4 weeks; and G361, 2.0 μg/ml for 6 weeks), and maintained in their respective media with 1 μg/mL puromycin.

### Forced Overexpression of SIRT3

For SIRT3 forced overexpression, as described earlier (19), bacterial glycerol stab with SIRT3-Flag plasmid (Addgene) was streaked onto agar plates containing ampicillin and incubated overnight at 37°C. Next day, four single colonies were picked and inoculated in LB medium. For SIRT3 overexpression, Hs294T cells were stably transfected with four isolated plasmid DNA of SIRT3-Flag and empty vector pcDNA 3.1(+) using Lipofectamine-2000 as per manufacturer’s instructions (Invitrogen). After 48 h, geneticin (2 mg/ml G-418; Gibco BRL) was added in the medium until antibiotic-resistant colonies developed. Individual colonies were isolated and propagated, and SIRT3-Flag-tagged protein was detected by immunoblot analysis. The colonies expressing the highest levels of SIRT3-Flag were selected for further analysis.

### Xenograft Studies

The xenograft experiments were performed under a protocol approved by the University of Wisconsin Animal Care and Use Committee. Female nude mice (Crl : NU-Foxn1nu) aged 6 weeks were procured from Charles River Laboratories. We conducted two sets of the experiment using twelve mice per experimental group. For SIRT3 knockdown, 2 x 10^6^ cells (shNS-G361 and shSIRT3-G361) and for SIRT3 overexpression, 1 x 10^6^ cells (Hs294T-pcDNA3.1 and Hs294T-SIRT3-Flag) mixed with Matrigel (BD Biosciences) at the ratio of 1:1 were injected subcutaneously on the right flank of each mouse. Once tumors were palpable, they were measured twice per week (Hs294T-xenografts) or weekly (G361-xenografts) using digital calipers and tumor volumes were calculated according to the formula: 0.52 (height x length x width). Hs294T-xenografted mice were sacrificed when tumors reached 20 mm in any one dimension, in any group. However, a 10 mm cut-off point was selected for G361-xenografted mice due to slow-growing tumors in these mice.

### RNA Isolation, Quantitative Real-Time PCR (RT-qPCR) and Glucose Metabolism PCR Array Analyses

RNA from cell lines and tissue samples were isolated using an RNeasy plus mini kit (Qiagen) as per the manufacture’s protocol. Corresponding cDNAs were synthesized using Oligo DT primers, dNTPs and M-MLV reverse transcriptase (Promega). RT-qPCR analyses were performed as described earlier using SYBR Premix Ex Taq II (TaKaRa) and QuantStudio 3 (ThermoFisher Scientific) (23). The primers for SIRT3, PCNA, Survivin, VEGF and ACTB were selected from the PrimerBank database (24). Relative transcript of each gene of interest was calculated using the ΔΔC_T_ method and ACTB as an endogenous control.

The human glucose metabolism PCR array (Qiagen #PAHS-006Z) was run using cDNA isolated from shNS SK-MEL-2 and shSIRT3 SK-MEL-2 cells, and analyzed as described earlier (23). Briefly, the resulting cycle threshold (CT) values were uploaded onto GeneGlobe Data Analysis Center (Qiagen) (http://www.qiagen.com/us/shop/genes-and-pathways/data-analysis-center-overview-page/) to analyze fold change in transcripts in response to SIRT3 knockdown. Three housekeeping genes, *ACTB, HPRT1* and *RPLP0*, were used to normalize the data. Genes from the PCR array showing ≥1.96-fold change with statistical significance were selected for further analysis. Three biological replicates were used to assess the levels of the 84 metabolism-related genes.

### Ingenuity Pathway Analysis (IPA)

To understand the pathways modulated in response to SIRT3 knockdown, differentially expressed genes from PCR array were uploaded on the IPA web portal (Qiagen; www.ingenuity.com). The data were analyzed to predict gene interaction network, cumulative functions, canonical pathways and upstream regulators for SIRT3-modulated genes.

### Quantitative Immunodetection Analysis Using ProteinSimple

Protein from tumor tissues was isolated in 1X RIPA lysis buffer (Millipore), and quantified using Pierce BCA Protein assay kit (Thermo Scientific) as described earlier (25). Wes ProteinSimple, a capillary-based immunodetection system was used for protein analysis. Briefly, as per the manufacturer’s protocol, Dithiothreitol, fluorescent 5× master mix, biotinylated ladder and luminol-peroxide solution were prepared. One part 5× fluorescent master mix was combined with 4 parts of protein lysate (0.5 µg/ul) in a microcentrifuge tube, and heated at 95°C for 5 min to denature the protein. These prepared proteins were loaded on 12–230 kDa Wes Separation Module (ProteinSimple), along with biotinylated ladder, antibody diluent (blocking reagent), primary antibodies, horseradish peroxidase (HRP)-conjugated secondary antibodies, streptavidin-HRP, luminol peroxide solution, and wash buffer, into the designated compartments. The primary antibodies were optimized and used at 1:50 dilution for VEGF (Proteintech #19003-1-AP), SIRT3, PCNA and Survivin (Cell Signaling #2627, 2586 and 2808, respectively). The produced chemiluminescence was detected at multiple exposure times and automatically quantified by the Compass for Simple Western software. The electropherograms showing automatically detected peak area for PCNA, Survivin or, VEGF were quantified and normalized to the total capillary area of the Total Protein Assay of the same sample.

### Statistical Analysis

Statistical analyses were performed using GraphPad Prism 5 software. The statistical test applied for each data is indicated in their respective figure legends. Data are expressed as mean ± SEM of three replicates, and statistical significance is denoted as *p<0.05, **p<0.01, ***p<0.001, ****p<0.0001.

## Results

### Tumorigenic Behavior of Genetically Manipulated Melanoma Cells for SIRT3 in Nu/Nu Mice Was Consistent With the Pro-Proliferative Role of SIRT3 in Melanoma

**Figure 1:**
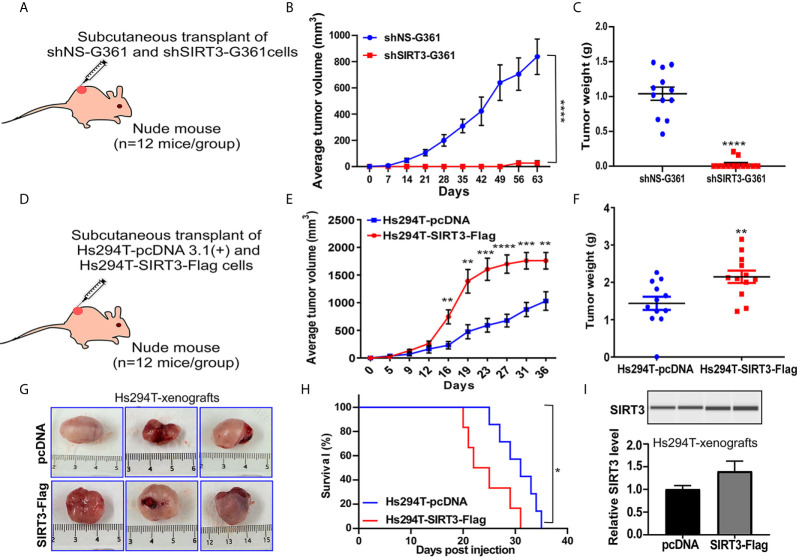
Effect of SIRT3-knockdown (shSIRT3-G361) and overexpressing (Hs294T-SIRT3-Flag) melanoma cells in Nu/Nu mouse xenografts. SIRT3-knockdown and overexpressing melanoma cells were xenografted in Nu/Nu mice and followed for tumorigenesis. **(A)** SIRT3-knockdown study design, **(B)** Average tumor volume, and **(C)** Tumor weight for shNS-G361 and shSIRT3-G361 melanoma cells xenografted tumors (at the termination of the experiment). **(D)** SIRT3- overexpression study design, **(E)** Average tumor volume, **(F)** Tumor weight (at the termination of the experiment), **(G)** Representative images of tumors with scale bars, and **(H)** Survival probability analysis for Hs294T-empty vector pcDNA 3.1(+) (control) and Hs294T-SIRT3-Flag (SIRT3-overexpressing) melanoma cells xenografted tumors. **(I)** Wes ProteinSimple analysis confirming SIRT3 higher expression in the derived tumor tissues. The SIRT3 quantitative data was normalized using Total Protein Assay of the same sample. N=12 mice/experimental group. Statistical significance are indicated as *p < 0.05, **p < 0.01, ***p < 0.001, ****p < 0.0001. shNS, nonspecific shRNA; shSIRT3, SIRT3-knockdown; pcDNA (empty vector); SIRT3-Flag (SIRT3-overexpression).

We have previously demonstrated that SIRT3 knockdown in NRAS-mutant SK-MEL-2 human melanoma cells inhibited tumor growth in a subcutaneous implanted Nu/Nu nude mouse model (19). Here, we expanded our work to confirm our previous results of SIRT3 knockdown in BRAF-mutant human melanoma cell line G361. For this purpose, Nu/Nu nude mice were subcutaneously implanted with shNS-G361 (control) and shSIRT3-G361 (SIRT3 knockdown) melanoma cells followed by assessing tumor development and progression (**Figure 1A**). Every mouse that received shNS-G361 developed a tumor approaching ~1000 mm^3^ in volume by 56 days (**Figure 1B**). However, only 2 mice out of 12 implanted with shSIRT3-G361 cells developed extremely small size tumors measuring 150 mm^3^ and 40 mm^3^ respectively. At the termination of the study, we weighed wet tumors and found significant inhibition in tumor weight, as well. These results suggest that SIRT3 knockdown resulted in a significant effect in tumorigenicity of G361 cells, with almost complete inhibition of tumor growth in mice xenografts (**Figures 1B, C**).

The pro-proliferative role of SIRT3 was also shown in the xenograft experiment with SIRT3 overexpressing melanoma cells in Nu/Nu nude mice. We have earlier demonstrated that SIRT3 overexpression promotes the proliferative potential of Hs294T melanoma cells, *in vitro* (19). In this study, we determined the tumorigenic potential of SIRT3 overexpressing Hs294T cells in Nu/Nu mice. The mice were subcutaneously implanted with Hs294T-empty vector pcDNA 3.1(+) (control) and Hs294T-SIRT3-Flag (SIRT3 overexpressing) melanoma cells and tumorigenicity of the cells was followed (**Figure 1D**). We found that SIRT3 overexpressing melanoma cells demonstrated significant increase in tumorigenicity, measured by both tumor volume and weight (**Figures 1E, F**). Representative images of resected tumors are shown in **Figure 1G**. Further, the Kaplan-Meier analysis showed that SIRT3 overexpression resulted in a significant survival detriment, in terms of reaching the tumor cutoff size 20 mm in one dimension (**Figure 1H**). SIRT3 expression was assessed in isolated tumor tissues and found a marked increase in SIRT3-Flag xenografts (**Figure 1I**). Though exogenous forced overexpression of SIRT3 may not represent physiologic phenomena as it generates supraphysiological SIRT3 levels, our results support the pro-proliferative function of SIRT3 in melanoma, further validating our earlier study (19).

### Genetic Manipulation of SIRT3 Caused Modulations in Markers of Proliferation, Survival and Angiogenesis in Melanoma Xenografts

**Figure 2:**
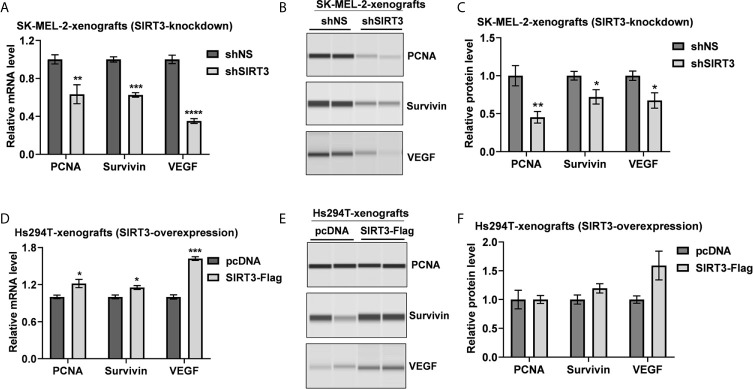
Effect of SIRT3-knockdown and overexpression on the key tumor markers related to cell proliferation, survival and angiogenesis. Utilizing tumor tissues from SIRT3-knockdown (shSIRT3- SK-MEL-2) melanoma cell xenografts, **(A)** RT-qPCR and **(B)** Wes ProteinSimple analyses were performed for PCNA, Survivin and VEGF mRNA and protein levels. Next, utilizing tumor tissues from SIRT3-overexpressing (Hs294T-SIRT3-Flag) melanoma cell xenografts, **(C)** RT-qPCR and **(D)** Wes ProteinSimple analyses were performed for PCNA, Survivin and VEGF mRNA and protein levels. ACTB was used as an endogenous control for transcripts analysis. The protein quantitative data were normalized to the total capillary area of the Total Protein Assay of the same sample. Data are presented as the mean ± SEM of three biological pools (n = 6) with statistical significance (multiple t-test, *p < 0.05, **p < 0.01, ***p < 0.001, ****p < 0.0001). shNS, nonspecific shRNA; shSIRT3, SIRT3-knockdown; pcDNA (empty vector); SIRT3-Flag (SIRT3-overexpression).

Due to an almost complete inhibition of tumor growth in shSIRT3-G361 implanted tumor group, we did not have sufficient tumor tissues from this group for further analyses. Therefore, we used archival tumor samples from our previous study, which utilized SIRT3 knockdown in SK-MEL-2 melanoma cells xenografted in Nu/Nu mice (19). We evaluated the effects of SIRT3 manipulation on markers of cell proliferation (PCNA), survival (Survivin) and angiogenesis (VEGF) using RT-qPCR and Wes ProteinSimple analyses. As shown in **Figure 2A**, significant decreases in PCNA, Survivin and VEGF transcripts were observed in SIRT3 knockdown tumor tissues. Similar results were noticed at the protein level for PCNA, Survivin and VEGF in SIRT3 knockdown tumor tissues (**Figures 2B, C**). We also analyzed the levels of PCNA, Survivin and VEGF transcripts and protein in SIRT3 overexpressing (Hs294T-SIRT3-Flag) tumor tissues. Our analysis found marked increase in PCNA, Survivin and VEGF transcripts in SIRT3 overexpressing tumor tissues (**Figure 2D**). However, at the protein level, a marked increase in VEGF and a marginal increase in Survivin were observed (**Figures 2E, F**). No change in PCNA was found probably due to the high background because of the higher number of PCNA positive proliferating cells in tumors. Overall, these data are consistent with the pro-proliferative role of SIRT3 in melanoma.

### SIRT3 Knockdown Altered the Expression of Key Metabolic Genes in Human Melanoma Cells *In Vitro*

**Figure 3:**
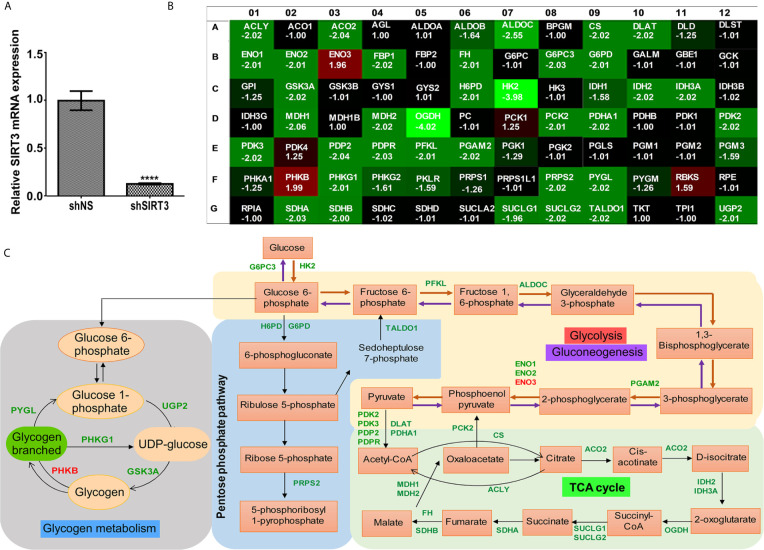
Effect of SIRT3-knockdown on metabolism-related gene expression in SK-MEL-2 human melanoma cells. **(A)** RT-qPCR analysis confirming SIRT3 knockdown in SK-MEL-2 cells. ACTB was used as an endogenous control. Data is presented as mean ± SEM (t-test, ****p < 0.0001). **(B)** Utilizing cDNA from SIRT3-knockdown (shSIRT3- SK-MEL-2) melanoma cell, Glucose metabolism PCR array was run and analyzed to assess the expression levels of 84 metabolism-related genes. Heat map of average gene expression in shSIRT3 SK-MEL-2 cells compared to shNS- SK-MEL-2 (control) is presented as fold change regulation. An increase in gene expression is depicted in red, whereas a decrease in gene expression is represented by green color. No differences in expression are depicted in black. **(C)** Significantly modulated genes showing ≥1.96-fold regulation were distributed accordingly to their contribution in different metabolic pathways. Fold changes were calculated using the ΔΔCt method. The values represent the average fold change of three independent experiments.

Given the fact that SIRT3 plays important role in cellular metabolism (4, 5), and has been identified as a key player in promoting cancer metabolism and tumor growth (26), we investigated the effect of SIRT3 knockdown on genes involved in energy uptake metabolism in melanoma, employing a glucose metabolism PCR array containing 84 metabolism-related gene primers. shRNA-mediated knockdown of SIRT3 in SK-MEL-2 melanoma cells (**Figure 3A**) resulted in alteration in a number of metabolism-related genes (**Figure 3B**). For the analysis, we considered genes that were differentially expressed at least 1.96-fold when compared with the control cells (shNS-SK-MEL-2) and with P <0.05. We set the cut-off as 1.96 instead of 2 to include 3 more genes that were critical for the analysis. The next lowest cut-off point was 1.64-fold, which was not considered for analysis. The identified genes are depicted on metabolic pathways (**Figure 3C**). SIRT3 silencing significantly modulated genes related to glycolysis (ALDOC, ENO1, ENO2, ENO3, HK2, PFKL, PGAM2), gluconeogenesis (G6PC3, PCK2), Krebs cycle (ACLY, ACO2, CS, DLAT, FH, IDH2, IDH3A, MDH1, MDH2, OGDH, PCK2, PDHA1, SDHA, SDHB, SUCLG1, SUCLG2), and Pentose phosphate pathway (G6PD, H6PD, PRPS2, TALDO1) (**Figures 3B, C**). We also found SIRT3 knockdown-mediated alterations in the genes involved in the regulation of glucose metabolism (PDK2, PDK3, PDP2, and PDPR). Further, we observed significant changes in the genes involved in glycogen metabolism pathway (glycogen synthesis (UGP2), glycogen degradation (PYGL), and regulation of glycogen metabolism (GSK3A, PHKB, PHKG1) upon SIRT3 knockdown (**Figures 3B, C**). Collectively, these results suggest that inhibition of SIRT3 modulates cellular metabolism that may ultimately lead to an anti-proliferative response in melanoma cells.

### Functions Annotation of Glucose Metabolism Genes Identified in Response to SIRT3 Knockdown Predicted Cumulative Action Against Melanoma Cell Survival

**Figure 4:**
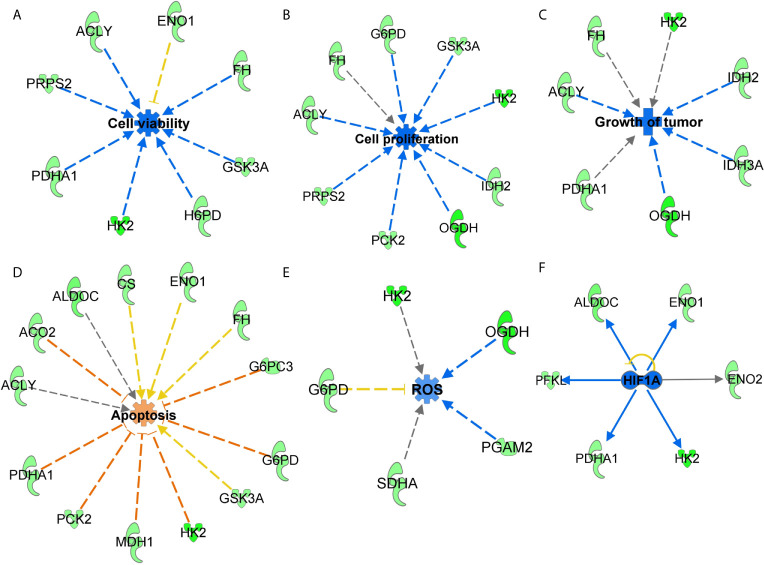
Functions annotation of glucose metabolism genes identified in response to SIRT3 knockdown (shSIRT3- SK-MEL-2). The cumulative effect of SIRT3 modulated genes were analyzed using IPA and effects were predicted for **(A)** cell viability, **(B)** cell proliferation, **(C)** tumor growth, **(D)** apoptosis and **(E)** ROS. Genes from the array are in green (downregulated), while predicted functions and interaction lines are in blue (inhibition) or orange (activation). Yellow lines indicate inconsistent findings, and gray dotted lines show unpredicted effects. **(F)** Using IPA, upstream regulator analysis predicted inhibition of HIF1α. Though other upstream regulators are presented in Figure 6, HIF1α inhibition is presented here to discuss SIRT3-HIF1α-ROS connections in cancer metabolism.

Next, to understand the effects of SIRT3-modulated metabolic genes, significantly altered genes were analyzed with IPA to predict their cumulative actions. Our analysis predicted the inhibition of cell viability, cell proliferation, tumor growth, and increased apoptosis (**Figures 4A–D**) in response to SIRT3 knockdown, which is in agreement with our *in vivo* findings. Though there are certain genes whose fold regulation with downstream effects are not consistent (yellow dotted lines) or predicted (gray dotted line), the cumulative effect remains the same. IPA also predicted a decrease in the generation of reactive oxygen species (ROS) in response to SIRT3 knockdown (**Figure 4E**). It is important to mention here that melanoma cells generate high ROS as a consequence of distorted melanosome structure (27), and thus, predicted inhibition of ROS supports the antitumor effect in response to SIRT3 inhibition in melanoma. The interaction of SIRT3 with hypoxia-inducible factor 1-alpha (HIF1α) has been shown to affect ROS homeostasis and glycolysis (28). Interestingly, the upstream analysis of SIRT3-modulated genes identified in our PCR array predicted the inhibition of HIF1α (**Figure 4F**), further supporting the involvement of SIRT3-HIF1α-ROS connection in cancer metabolism.

### Gene Network and canonical Pathway Analyses Showed Complex Interactions Among SIRT3 Modulated Genes and With Several Melanoma-Associated Genes

**Figure 5:**
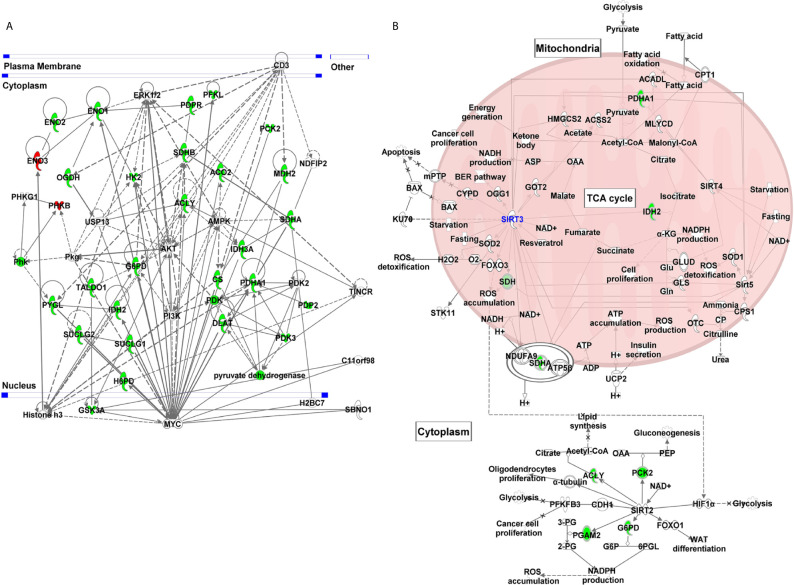
Gene network and canonical pathway analyses of glucose metabolism genes identified in response to SIRT3 knockdown (shSIRT3- SK-MEL-2). Differentially expressed genes from glucose metabolism PCR array with cut-off criteria of ≥1.96-fold change with statistically significant change were analyzed using IPA. **(A)** Gene network indicating complex interactions among SIRT3 modulated genes and with several cancer/melanoma-associated genes. **(B)** Canonical pathway showing the interaction of SIRT3 with some of the SIRT3-modulated genes along with other sirtuins. Genes are represented with green (downregulated) or red (upregulated) or no color (appeared during gene network analysis). Solid lines indicate robust interactions, and dashed lines are significant but less frequent.

We employed IPA to explore the gene network and canonical pathway associated with SIRT3-modulated genes. Our analysis identified a gene network with 29 focus molecules (out of 37) indicating that these genes in the network were systematically associated (**Figure 5A**). The network pathway analysis of interacting genes predicted links to various other important genes (denoted with uncolored nodes). IPA exploration of the gene network indicated the connection of SIRT3 modulated genes along with several melanoma-associated genes (MYC, PI3K, AKT, CD3, ERK, and AMPK). Moreover, oncogenic MYC appeared as a central molecule interacting with most of the SIRT3-modulated genes and the molecules that appeared during network generation.

Further, IPA predicted five canonical pathways suggesting that these signaling pathways were constitutively affected in response to SIRT3 knockdown. These included Krebs cycle, glycolysis, gluconeogenesis, and pentose phosphate pathway. Interestingly, sirtuin signaling pathway appeared too as a canonical pathway (**Figure 5B**). SIRT3 was found to have interactions with several mitochondrial signaling molecules along with PDHA1, IDH2 and SDHA, identified in PCR array. SIRT3 also showed interaction with other mitochondrial sirtuins viz. SIRT4 and SIRT5. Interestingly, SIRT2, a cytoplasmic sirtuin was predicted to directly interact with ACLY, PCK2, PGAM2 and G6PD, which were also identified in PCR array, suggesting SIRT2/SIRT3 connection with cellular metabolism. Intriguingly, SIRT2 is also known to contribute in melanoma progression (29).

### Upstream Analysis of SIRT3-Modulated Genes Predicted Alteration in Major Cancer/Melanoma-Associated Signaling Events

**Figure 6:**
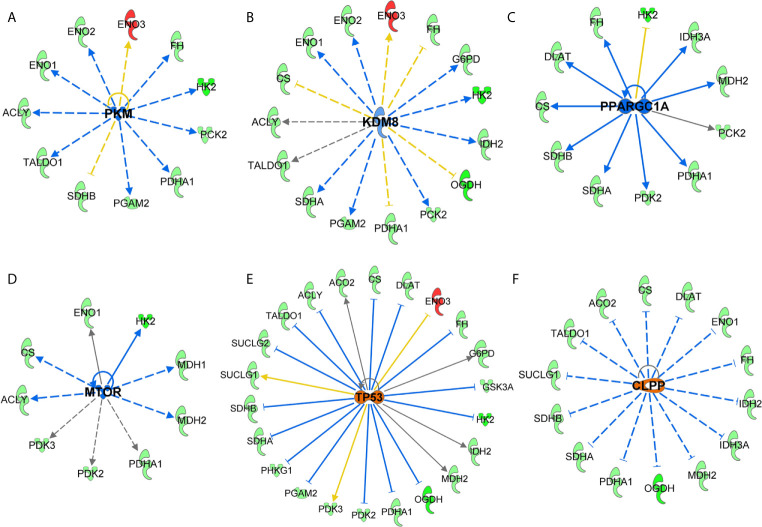
Key upstream signaling pathways predicted in response to SIRT3 knockdown (shSIRT3- SK-MEL-2). Using IPA, upstream regulator analysis identified genes potentially involved in melanoma or cancer. These are **(A)** PKM, **(B)** KDM8, **(C)** PPARGC1A, **(D)** mTOR, **(E)** P53, and **(F)** CLPP signaling. Upstream regulations are denoted with blue (inhibition) **(F)** or orange (activation) color. The interaction lines are indicated as solid (robust interactions), dashed (significant but less frequent), blue (inhibition), orange (activation), yellow (finding inconsistent), and gray (unpredicted).

Next, we performed the upstream analysis of SIRT3-modulated genes and identified alteration in several crucial upstream regulators that are known to affect cancer/melanoma development and progression. Specifically, our analysis predicted inhibition in pyruvate kinase muscle (PKM) isozyme, lysine demethylase 8 (KDM8), peroxisome proliferator-activated receptor gamma-coactivator 1 alpha (PPARGC1A) and mammalian target of rapamycin (mTOR) signaling (**Figures 6A–D**). Our analysis further predicted the activation of tumor suppressor P53 and mitochondrial caseinolytic protease P (CLPP) signaling (**Figures 6E, F**). Inhibition in HIF1α was also predicted (which has been discussed earlier in **Figure 4F**). Our data shows that most of the SIRT3 modulated genes were consistent with the state of upstream prediction. Those with upstream predictions in-consistent or un-known are indicated with yellow or gray dotted lines, respectively. The relevance of modulations in these upstream regulators related to cancer/melanoma development and progression are detailed in the discussion section. Overall, these are important observations to demonstrate a crucial connection between SIRT3 and key cancer/melanoma-associated signaling events.

## Discussion

The goal of this study was to validate the pro-proliferative function of SIRT3 in melanoma and establish its associated potential mechanisms, especially focusing on metabolic regulation. Our study includes data from BRAF-mutant G361 and Hs294T as well as NRAS-mutant SK-MEL-2 melanoma cells. These two mutations (BRAF and NRAS) are the most common genetic alterations in human melanoma (30). We investigated the tumorigenic behavior of SIRT3 knockdown and overexpression in melanoma cells in Nu/Nu mice. Our data demonstrated that SIRT3 knockdown abrogated tumor growth and/or tumor establishment abilities in BRAF-mutant G361-xenografted mouse model. Also, forced overexpression of SIRT3 in BRAF-mutant Hs294T-xenografted mouse model promoted tumorigenesis in melanoma. These data along with our previous study of SIRT3 knockdown in NRAS-mutant SK-MEL-2-xenografted mouse model (19) support the pro-proliferative nature of SIRT3. This also suggests that antitumor effects observed in response to SIRT3 inhibition are independent of BRAF or NRAS mutations.

Utilizing RT-qPCR and Wes ProteinSimple analyses, we found modulation in the markers of cell proliferation (PCNA), survival (Survivin) and angiogenesis (VEGF) in response to SIRT3 manipulation, further supporting the pro-proliferative role of SIRT3 in melanoma. These are important observations from the following perspectives too. Cytoplasmic PCNA has recently been shown to control glucose metabolism in hematological cells (31). Survivin has been shown to increase the stability of oxidative phosphorylation Complex II, which enhances mitochondrial respiration, and thereby cancer metabolism (32). Proangiogenic factor VEGF has been reported to be an indicator of angiogenesis in melanoma (33). It is known that the tumor cells undergo metabolic changes during angiogenesis that favor tumor growth and progression. Our data suggest the melanoma promoting potential of SIRT3 may be linked with alteration in the metabolic phenotypes, and thus SIRT3 inhibition may be a potential strategy to inhibit melanoma progression.

As SIRT3 has been implicated in regulating cellular metabolism, we determined the role of SIRT3 in metabolic regulation of melanoma cells. Melanoma cells are known to display metabolic adaptations with deregulated glycolysis that favors tumorigenesis (34). Earlier, we have demonstrated that chemical inhibition of SIRT3 along with SIRT1 decreased aerobic glycolysis (glucose uptake, lactate production and NAD+/NADH ratio) in melanoma cells (35). Importantly, the inhibition of glycolysis in cancer has been suggested as a potential target for cancer therapy. Thus, to analyze the effects of SIRT3 knockdown on metabolic regulators in melanoma cells, we utilized glucose metabolism PCR array. We found that SIRT3 inhibition significantly modulates genes related to glycolysis, gluconeogenesis, Krebs cycle and Pentose phosphate pathway, suggesting that inhibition of SIRT3 hampers cellular metabolism, which eventually hinders the proliferative potential of melanoma cells. This can be understood from the fact that tumor cells require not only ATP but also biosynthetic precursors for proliferation. Glycolysis, Pentose phosphate pathway and Krebs cycle are the important source of biosynthetic precursors, in addition to the production of ATP. For example, citrate in mitochondria can be transported into cytoplasm for the conversion of acetyl-CoA and oxaloacetate to fatty acids, which is critical for tumor cell proliferation. This conversion is controlled by the enzyme ATP citrate lyase (ACLY) (36), which was decreased in response to SIRT3 inhibition. The expression levels of glycolytic enzymes, enolase 1 and 2 (ENO1/2) were also lesser in cells having reduced SIRT3, suggesting the metabolic cessation of glycolytic pathway. Enolases are key components of the glycolytic pathway, converting 2-phosphoglycerate into phosphoenolpyruvate. ENO1 knockdown has been shown to result in suppressed glioma cell growth (37). Also, increased ENO2 has been shown to be associated with tumor progression *in vivo* (38). However, ENO3, which is muscle-specific enolase, was increased in response to SIRT3 knockdown. This may be compensatory for the downregulation of its counterpart ENO1 and ENO2, suggesting that the role of enolases needs to be explored in melanoma.

Our data demonstrated that hexokinase (HK2), and oxoglutarate (alpha-ketoglutarate) dehydrogenase (OGDH) were the top two genes that showed maximum changes in the PCR array in response to SIRT3 knockdown. HK2 phosphorylates glucose into glucose-6-phosphate, which serves as the start point for glucose to enter glycolytic pathway and mitochondrial Krebs cycle to produce ATP (39). Aside from being a fundamental component in glycolysis, HK2 is over-expressed in multiple tumors (40). Similarly, in a recent study, cancer cells have been found to display a wide range of sensitivities in response to OGDH knockdown, *in vitro* and *in vivo* (41), suggesting a probable therapeutic target in cancer management. Next, we found that the isoforms of pyruvate dehydrogenase (PDH) complex (PDHA1, PDP2, PDK2 and PDK3), which constitute the link between glycolysis and Krebs cycle (42), were down-regulated upon SIRT3 inhibition, indicating reduced substrate availability for cells to enter the Krebs cycle. Interestingly, SIRT3 is known to deacetylate and increase PDH activity in cancer cells (22). SIRT3 is also known to deacetylate mitochondrial enzyme, isocitrate dehydrogenase (IDH2) that produces NADPH (43); IDH2 was suppressed too upon SIRT3 inhibition. We also found that both glucose-6-phosphate dehydrogenase (G6PD) and hexose-6-phosphate dehydrogenase (H6PD) were significantly down-regulated in SIRT3 knockdown cells, suggesting inhibition of the Pentose phosphate pathway. Overall these results suggest that modulation in these genes in response to SIRT3 inhibition results in metabolic shift in melanoma cells providing lesser fuel for proliferating cells.

Next, upstream analysis of SIRT3 modulated genes predicted the inhibition of HIF1α, PKM, KDM8, PPARGC1A and mTOR, and activation of P53 and CLPP signaling. The relevance of these observations in melanoma can be understood from the following facts. PKM is known to regulate glycolysis in cells including in malignancies (44, 45). PKM has also been known to be related to the invasiveness of cancers. Indeed, PKM has been identified as one of the major hypoxia-induced HIF1α targets in melanocytes that significantly correlate with reduced melanoma disease-free status (46). Moreover, KDM8 which demethylates H3K36me2 (inhibiting the recruitment of histone deacetylases) and is overexpressed in different types of tumors (47), is known to participate in nuclear translocation of PKM. Similarly, upstream PPARGC1A is used by invasive cancer cells to enhance oxidative phosphorylation, oxygen consumption rate, and mitochondrial biogenesis (48). Oxidative phosphorylation has been shown to promote primary melanoma invasion, suggesting the possible role of PPARGC1A in metabolic rewiring during melanoma progression (49). mTOR, which acts as the target for cell-cycle arrest, is another important signaling predicted to be inhibited in response to SIRT3 inhibition. mTOR deregulation has been observed in many cancer types, including melanoma, and its inhibition has been investigated in clinical treatment (50). Moreover, its association with phosphatidylinositol 3-kinase (PI3K)/protein kinase B (AKT) pathways to regulate cell growth, survival and metabolism make a target of interest in cancer management (50). Interestingly, in our study, we identified the connections of PI3K/AKT and mTOR in response to SIRT3 inhibition (**Figures 5A** and **6D**).

Our analysis of SIRT3-modulated genes further identified upstream activation of tumor suppressor P53. An interaction between SIRT3 and tumor suppressor P53 has been shown in certain cancers. SIRT3 partially abrogates P53 activity to enact growth arrest and senescence in bladder carcinoma (51). Earlier, we have demonstrated that inhibition of SIRT1, another member of sirtuin family, decreased cell proliferation of melanoma cells *via* P53 activation (52, 53). We have also shown a protein network highlighting P53 as key signaling connecting with MYC, and other proteins in response to SIRT1 inhibition (54). SIRT1 and SIRT3 both have been demonstrated to deacetylate P53 protein, and the role of P53 has been implicated in melanoma (reviewed in (55)). Activation of CLPP, which is known to degrade misfolded proteins, was also predicted in our IPA analysis in response to SIRT3 knockdown. Interestingly, it has been found that hyperactivation of CLPP selectively kills cancer cells, without affecting normal cells, and independent of P53 status, by disrupting mitochondrial structure/function *via* the degradation of respiratory chain protein substrates (56).

Overall, our study identified the involvement of SIRT3 in altering the metabolic phenotypes in melanoma. Specifically, our data suggest that modulation of SIRT3 affects the growth of melanoma xenografts as well as various tumor growth markers. Further, our data suggest that inhibition of SIRT3 reverses the glycolytic shift *via* down-regulating key metabolic genes. However, further studies are required to validate SIRT3 as a therapy target in suitable genetically engineered and/or patient-derived xenografts (PDX) models of melanoma. Given the important role of SIRT3 in metabolism, there is always an issue of off-target complications. Therefore the use of SIRT3 as a potential target for melanoma management needs to be carefully investigated.

## Data Availability Statement

The original contributions presented in the study are included in the article/supplementary material. Further inquiries can be directed to the corresponding author.

## Ethics Statement

The animal study was reviewed and approved by University of Wisconsin Animal Care and Use Committee.

## Author Contributions

Conception and design of the study: CS, JG and NA. Methodology: CS, JG, GC, MN. Analysis and interpretation of the data: CS, JG, GC, MN, HC and NA. Writing - original draft preparation: CS. Writing, review and editing: CS, JG, GC, MN, HC and NA. All authors contributed to the article and approved the submitted version.

## Funding

This work was partially supported by funding from the NIH (R01AR059130 and R01CA176748), and the Department of Veterans Affairs (VA Merit Review Awards I01BX001008 and I01CX001441; and a Research Career Scientist Award IK6BX003780). We also acknowledge the core facilities supported by the Skin Diseases Research Center (SDRC) Core Grant P30AR066524 from NIH/NIAMS.

## Conflict of Interest

The authors declare that the research was conducted in the absence of any commercial or financial relationships that could be construed as a potential conflict of interest.
